# Interrogating the Glycolytic Axis in Pulmonary Hypertension Models: Potential Targets for Therapy

**DOI:** 10.1002/pul2.70444

**Published:** 2026-10-30

**Authors:** Rachel L. Sutcliffe, Iona Cuthbertson, Stephen Moore, Mark Southwood, Laura de La Bastida Casero, Benjamin Dunmore, Stefan J. Marciniak, Paul Upton, Nicholas Morrell, Paola Caruso, Elaine Soon

**Affiliations:** ^1^ Department of Medicine University of Cambridge Cambridge UK; ^2^ MRC Toxicology Unit Cambridge UK; ^3^ Centro de Investigaciones Biologicas “Margarita Salas” Madrid Spain; ^4^ Cambridge Institute for Medical Research University of Cambridge Cambridge UK

**Keywords:** glycolysis, PKM2, pulmonary arterial hypertension, TEPP‐46

## Abstract

Pulmonary artery ECs from PAH patients display reduced oxidative phosphorylation and increased anabolic and pro‐proliferative effects (“Warburg phenomenon”). TEPP‐46 (allosteric stabilizer of tetrameric PKM2) has beneficial effects in wild‐type and BMPR2‐mutation bearing BOECs, ameliorates the increased right ventricular systolic pressures, and improves survival in Sugen‐hypoxia‐exposed rats. Antagonizing the Warburg effect may offer novel therapeutic options for PAH.

## 1 Introduction

Pulmonary arterial hypertension (PAH) describes diseases characterized by high pulmonary pressures (> 20 mmHg) [1] that lead to right ventricular failure and eventually death. This is due to remodeling of small‐to‐medium pulmonary vessels, with eventual lumen obliteration. PAH development is multifactorial, with multiple “hits” required for initiation and pathogenesis, akin to carcinogenesis. Mutations in *BMPR2*, pro‐inflammatory states, and endothelial cell metabolic abnormalities [2] have all been implicated as contributory factors.

Healthy endothelial cells are able to rapidly shift from quiescence to proliferative and apoptotic states, partly due to a greater utilization of glycolysis for ATP production, rather than the slower oxidative phosphorylation more commonly relied on by other cell types [3]. In PAH patients, pulmonary artery endothelial cells demonstrate an even greater shift towards glycolytic ATP production [4]. This phenomenon, first described in cancer as the “Warburg effect,” is considered to be an adaptation for optimizing both energy production and biosynthetic processes for maximum proliferative potential [5].

Pyruvate kinase plays a critical role in glycolysis, as this enzyme catalyzes the conversion of phosphoenolpyruvate to pyruvate, which then either fuels oxidative phosphorylation, through the citric acid cycle, or is converted to lactate by lactate dehydrogenase A (LDHA). There are four pyruvate kinase isoforms. PKM1 and PKM2 are the predominant isoforms in endothelial cells and arise from alternative splicing– inclusion of exon 9 results in PKM1, and inclusion of exon 10 results in PKM2. PKM2 is mainly expressed in embryonic cells and is progressively replaced by PKM1 in adult tissue.

PKM2 shifts between a highly enzymatically active homotetrameric form, which converts phosphoenolpyruvate into pyruvate, and a less active homodimeric form, which can translocate to the nucleus and regulate the expression of numerous proteins, which promote proliferative and glycolytic processes (Figure 1A) [6]. Due to the limited enzymatic activity of dimeric PKM2, this isoform, when prevalent, slows the production of pyruvate, leading to build‐up of glycolytic intermediates that are redirected into anabolic pathways.

**Figure 1:**
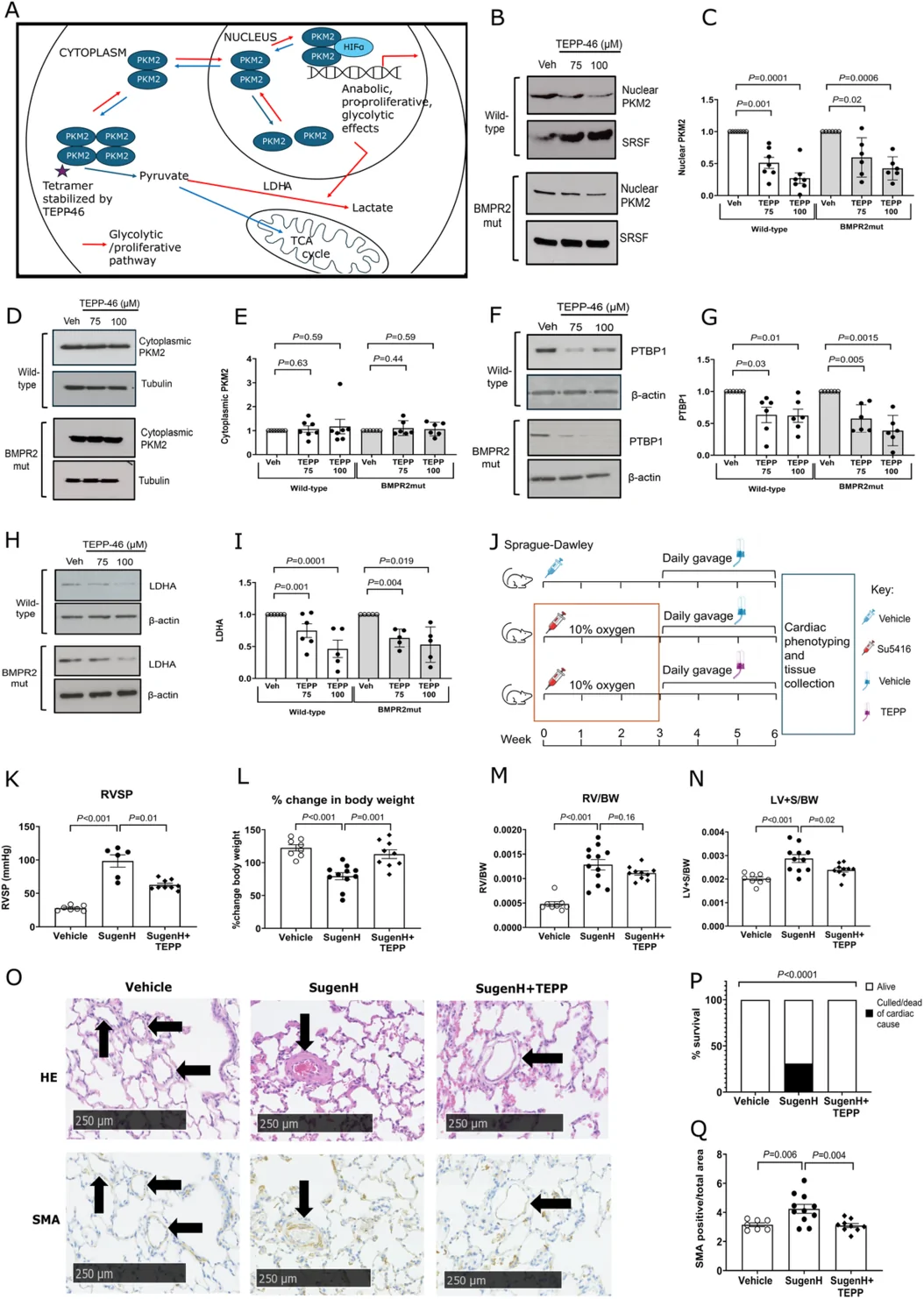
Characterization of how TEPP‐46 affects the PKM2 axis in BOECs and the Sugen‐hypoxia rat model. (A) Shows a PKM2 axis schematic. (B, C) Show a representative western blot analysis for nuclear PKM2 (B) in wild‐type and *BMPR2‐*mutant BOECs after 48 h of treatment with either vehicle, 75 µM, or 100 µM of TEPP‐46; and densitometry (C). (D, E) Show a representative Western blot analysis of corresponding cytoplasmic PKM2 (D) levels after treatment with either vehicle, 75 µM, or 100 µM of TEPP‐46; and densitometry (E). (F−I) Show PTBP1 and LDHA levels in wild‐type and *BMPR2‐*mutant BOECs after 48 h of treatment with either vehicle or TEPP‐46. (F) and (H) Show representative western blot analysis and (G) and (I) the corresponding densitometry. (J) Summarizes the Sugen‐hypoxia protocol. (K, L) Show the right ventricular systolic pressures (RVSP, K); the change in body weight (L), the right ventricular weight indexed to body weight (RV/BW, M), and the left ventricular and septum weight indexed to body weight (LV + S/BW, N) of these rats. (O) Shows serial rat lung sections from each cohort, stained for hematoxylin and eosin (HE) or α‐smooth muscle actin (SMA). Black arrows show small blood vessels (≤ 250 micrometers). (P) Shows the survival of these animals as a percentage of their respective cohorts. (Q) Shows SMA quantification.

We hypothesize that targeting the PKM2 axis may shift the balance to favor oxidative phosphorylation, reversing the Warburg effect, and that this would be beneficial in cell and rodent PAH models. Therefore, we evaluated TEPP‐46, an allosteric PKM2 activator that stabilizes the tetrameric form, blocking nuclear translocation and increasing canonical enzymatic activity [7].

## 2 Materials and Methods

We selected blood outgrowth endothelial cells (BOECs) as our cell model as these closely mimic mature pulmonary artery endothelial cells, with only 0.005% of genes differentially expressed between them [8]. They were isolated from healthy volunteers' blood and from PAH patients bearing *BMPR2* mutations and cultured as previously described [8]. Sprague‐Dawley rats were sourced from Charles River (UK) and injected subcutaneously with the VEGFR inhibitor Sugen‐5416 (20 mg/kg) or vehicle prior to entering hypoxia (10% oxygen) or normoxia for 21 days. Following hypoxic or normoxic exposure, rats were randomly allocated to oral gavage with either vehicle or 50 mg/kg TEPP‐46 daily for 21 days. More information is available in the Methods and Supporting Information S1: Tables 1−3.

## 3 Results

Wild‐type and *BMPR2‐*mutant BOECs were treated with either vehicle or TEPP‐46 for 48 h. After TEPP‐46 exposure, nuclear PKM2 levels (i.e., the proliferation‐promoting homodimer) decreased (Figure 1B,C) while cytoplasmic levels (where both forms exist) remain unchanged (Figure 1D,E) in both genotypes. TEPP‐46 treatment reduced levels of polypyrimidine tract‐binding protein 1 (PTPB1), which controls alternative splicing of the *PKM* gene to promote PKM2 expression (Figure 1F,G); and lowered levels of LDHA, which converts pyruvate to lactate, preventing its utilization for oxidative phosphorylation (Figure 1H,I) in both wild‐type and *BMPR2‐*mutant BOECs.

We then decided to evaluate the effects of TEPP‐46 in the Sugen‐hypoxia rat model, which causes severe pulmonary hypertension mimicking human disease (Figure 1J [9]). Oral TEPP‐46 reduced the elevated right ventricular systolic pressures (Figure 1K) and ameliorated the body weight loss associated with Sugen‐hypoxia (Figure 1L). The decrease in the right ventricular mass indexed to body weight (Figure 1M) did not reach significance, although interestingly, the increase in the left ventricular mass index was ameliorated by TEPP‐46 (Figure 1N).

Immunohistochemical examination revealed increased muscularization of the smaller pulmonary vessels (50−200 μm) after Sugen‐hypoxia (Figure 1O). These changes were markedly improved by TEPP‐46, and this improvement was also associated with greater survival (Figure 1P). Exposure to Sugen‐hypoxia increased smooth muscle actin‐positivity and smooth muscle cell numbers, and this change was ameliorated by oral TEPP‐46 (Figure 1Q). Blood lactate levels were increased after exposure to Sugen‐hypoxia (Supporting Information S1: Figure 1A). The rise in blood glucose due to Sugen‐hypoxia did not reach significance (Supporting Information S1: Figure 1B); and TEPP‐46‐associated decreases in both lactate and glucose did not reach significance (note that the study was underpowered with respect to this, and it is estimated that 37 rats per treatment arm would be needed to examine this effect adequately).

## 4 Discussion

We show that pharmacological manipulation of the glycolytic process has beneficial effects in both wild‐type and BMPR2‐mutation bearing BOECs and a Sugen‐hypoxia rat model of PAH. Increased PKM2 expression has previously been shown in the right ventricles of rats subjected to pulmonary artery banding (PAB) and PAH patients. It is known that PARP1‐dependent poly‐ADP‐ribose (PAR) is critical to the nuclear retention and function of PKM2. Shimauchi et al. [10] showed that either genetic deletion of PARP1 or pharmacological blockade (olaparib, a PARP‐1 inhibitor; or TEPP‐46) was sufficient to improve markers of right ventricular function in rodents subjected to PAB.

Increased phosphorylated‐PKM2 levels (which prevent tetramerization) and decreased tetrameric forms of PKM2 have been shown in both lung tissue and pulmonary artery smooth muscle cells derived from PAH patients [11, 12]. In these pulmonary artery smooth muscle cells, TEPP‐46 inhibits hypoxia‐driven proliferation from both controls and PAH patients [12]. Our findings further substantiate the physiological and functional relevance of glycolysis in the PAH model. More comprehensive characterization of the metabolic phenotypes of both wild‐type and BMPR2‐mutant cells, using either Seahorse mitochondrial stress testing or Oroboros O2K‐based oxygen consumption assays, would provide further insight into the underlying metabolic mechanisms.

## 5 Conclusion

Our study findings, taken together with the published evidence, suggest that the Warburg effect is significant in multiple cell types and organs in PAH models and patients. We conclude that antagonization of this effect may offer a novel therapeutic option for this deadly disease.

## Author Contributions

Rachel Sutcliffe, Iona Cuthbertson, Stephen Moore, Laura de La Bastida Casero, and Mark Southwood performed experiments and analyzed data. Benjamin Dunmore, Stefan J. Marciniak, Paul Upton, and Nicholas Morrell helped conceptualize ideas, performed critical analysis, and provided supervision and funding. Paola Caruso and Elaine Soon analyzed data, wrote the manuscript, provided supervision and funding, and acted as guarantors. All authors read and revised the manuscript.

## Ethics Statement

This study was conducted in accordance with the Declaration of Helsinki. Human BOECs were isolated from healthy volunteers' and *BMPR2‐*mutation bearing PAH patients' blood [8], with approval from the East of England Research Ethics Review Committee (18/EE/0269). Animal procedures were performed in line with the Animals (Scientific Procedures) Act 1986 under the project license 70/8850(NWM).

## Conflicts of Interest

Paul Upton has been a consultant for Interact‐Bio Ltd. The other authors declare no conflicts of interest.

## Supporting information


Supporting File

## Data Availability

The data that support the findings of this study are available from the corresponding author upon reasonable request.
