# Supplementary material for: Interrogating the Glycolytic Axis in Pulmonary Hypertension Models: Potential Targets for Therapy
Source: Pulm Circ. 2026 Oct 30;16(4):e70444. doi: 10.1002/pul2.70444 (PMC13722308; doi:10.1002/pul2.70444)
Supplement: Supplementary file 1 [file PUL2-16-e70444-s001.docx]

**Online Supplement**

**Interrogating the glycolytic axis in pulmonary hypertension models: potential targets for therapy.**

Rachel Sutcliffe^1^, Iona Cuthbertson^1^, Stephen Moore^1^, Mark Southwood^1,2^, Laura de La Bastida Casero^3^, Benjamin Dunmore^1,4^, Stefan J.Marciniak^4^, Paul Upton^1^, Nicholas Morrell^1^, Paola Caruso^1,^*, Elaine Soon^1,4,^*

**Author Affiliations:**

^1^Department of Medicine, University of Cambridge; Cambridge CB2 0QQ, UK.

^2^MRC Toxicology Unit, Tennis Court Road; Cambridge CB2 1QR, UK.

^3^Centro de Investigaciones Biologicas ‘Margarita Salas’, Madrid, Spain.

^4^Cambridge Institute for Medical Research, University of Cambridge; Cambridge CB2 0XY, UK.

*equal contributions

**Supplemental Materials and methods**

*Cell culture and reagents*

Human BOECs were isolated from the peripheral blood of healthy volunteers and grown as previously described (Toshner *et al., Am J Respir Crit Care Med.* 2009;180(8):780-7), with approval from the Research Ethics Review Committee (18/EE/0269). TEPP-46 was the kind gift of Dr Craig Thomas. BOECs were seeded at the densities described in Table 1, grown to confluence, and treated with vehicle (EGM2 supplemented with 10% [v/v] FBS) + DMSO or TEPP-46 (75 or 100μM) in vehicle, for 48 hours, with fresh TEPP-46 treatment after 24 hours.

| **Culture plate/dish** | **Cell numbers** | **Media volume per well/plate (ml)** |
| --- | --- | --- |
| 6-well plate | 1.8-2x10^5^ | 2 |
| 60 mm dish | 3.9-4x10^5^ | 4.5 |

**Table 1: BOEC seeding densities.**

*Western blotting*

BOECs cultured in 60 mm dishes were lysed with 200μl of radioimmunoprecipitation assay (RIPA) buffer supplemented with cOmplete™ protease inhibitor cocktail (Emerald Scientific, 1:19 dilution) on dry ice. The dishes were gently scraped and lysate collected into 1.5ml Eppendorfs. Samples were sonicated for 25-30 seconds and centrifuged (15,000 x G, 15 minutes, 4^o^C) before the clarified supernatant (protein lysate) was collected. For certain experiments, nuclear and cytoplasmic fractions were obtained using the NE-PER™ nuclear and cytoplasmic extraction kit (78833, ThermoFisher Scientific, USA). Protein concentration in the clarified lysate was quantified using a Bicinchoninic acid (BCA) Protein Assay according to manufacturer’s instructions (Bio-Rad). Protein concentration was determined using linear regression of a standard curve of bovine serum albumin (BSA). Samples were aliquoted into 20-80μg stocks and stored at -80^o^C until use.

Protein samples were processed under reducing conditions, lysates were prepared for loading in laemelli buffer - 312.5 mM Tris-HCl, pH 6.8, 10% (w/v) sodium dodecyl sulphate, 50% (w/v) glycerol and 0.007% (w/v) bromophenol blue supplemented with 12.5% (v/v) β-mercaptoethanol to a 1:4 dilution; and heat-treated for 10 minutes at 95^o^C to achieve protein denaturation. Samples and PageRuler Plus Prestained Protein Marker (ThermoFisher Scientific) were loaded onto 12% polyacrylamide gels prepared in-house using National Diagnostics gel reagents or Novex NuPAGE™ 4-12% Tris-Glycine Gels (Invitrogen) in tanks containing SDS-running buffer. These gels were run at 100 V for 10 minutes, allowing samples to uniformly migrate through the lower density stacking gels into the higher density resolving gel, before running at 150 V for ~1.5 hours or until the desired ladder separation was achieved. For proteins with a molecular weight up to 100kDa, gels were transferred to 0.2μM pore sized polyvinylidene difluoride (PVDF) membranes using the P0 setting on the iBlot® 2 Dry Blotting System (ThermoFisher Scientific) or onto membranes by manual semi-dry transfer (Amersham PVDF, 0.2μM pore size, GE Healthcare) for ~1.5 hours, pre-activated by submersion in methanol. Proteins greater than 100kDa were transferred onto 0.2μM pore-sized PVDF membranes preactivated with methanol, via a wet-transfer system overnight (~16 hours) at 4^o^C and 90mA.

After completion of transfer, membranes were blocked with 5% (w/v) skimmed milk in Tris-buffered saline (TBS; 6.05 g/l Tris-Base,8 g/l NaCl, pH 7.4) supplemented with 0.05% (v/v) Tween 20 (TBST) for 1 hour at room temperature under gentle rotation. Blots were washed with TBS-T 3 times and incubated with the primary antibodies listed in Table 2. Membranes were also incubated with appropriate loading controls for 1 hour at room temperature before washing and addition of secondary antibodies. Membranes were washed again with TBS-T before incubation with 5% milk (v/v) containing appropriate streptavidin-conjugated secondary antibodies (Dako) for 1 hour at room temperature (1:2000, Table 3).

| **Target protein** | **Manufacturer** | **Reference** | **Blocking agent** | **Dilution** |
| --- | --- | --- | --- | --- |
| PKM2 | Cell Signaling, USA | 4053 | TBS-T, 5%(w/v) BSA | 1:1000 |
| PTBP1 | Abcam, UK | ab30317 | TBS-T, 5%(w/v) BSA | 1:1000 |
| LDHA | Cell Signaling, USA | 3582 | TBS-T, 5% (w/v) milk | 1:1000 |
| SRSF1 | Invitrogen, USA | 32-4600 | TBS-T, 5% (w/v) milk | 1:5,000 |
| Tubulin | Sigma-Aldrich, USA | T5168 | TBS-T, 5% (w/v) milk | 1:10,000 |
| Β-actin | Sigma-Aldrich, USA | A5441 | TBS-T, 5% (w/v) milk | 1:10,000 |

**Table 2: Primary antibodies used for Western blotting**

| **Target protein** | **Manufacturer** | **Code** | **Blocking agent** | **Dilution** |
| --- | --- | --- | --- | --- |
| Goat anti-rabbit | Dako, USA | P0448 | TBS-T, 5% (w/v) milk | 1:2000 |
| Goat anti-mouse | Dako, USA | P0447 | TBS-T, 5% (w/v) milk | 1:2000 |

**Table 3: Secondary antibodies used for Western blotting**

*Rat Sugen-hypoxia model*

All animal procedures were performed following University of Cambridge guidelines, and in line with the Animals (Scientific Procedures) Act 1986 under the project license 70/8850. Sprague-Dawley rats (~250-300g) were sourced from Charles River (UK) and allowed to acclimatize for 1 week. Power calculations were performed to determine the minimum group size required for sufficient statistical power (n=11, 20% effect change [1-sided] with 80% power and 95% confidence). Rats were randomly assigned to experimental groups and identified by tail number for experimental blinding purposes.

Rats were injected subcutaneously with the VEGFR inhibitor Sugen-5416 (20mg/kg) or vehicle prior to entering hypoxia (10% oxygen) or normoxia for 21 days. Following hypoxic or normoxic exposure, rats were randomly allocated to oral gavage with either vehicle or 50 mg/kg TEPP 46 (5 mg/ml v/v in 10% DMSO v/v, 20% HP-β-CD g/v) daily for 21 days. Blood lactate and glucose were measured directly from tail vein bleeds both prior to the initial oral gavage, and at the end of this period with lactate (using the EDGE blood lactate monitoring system, <https://www.edge-usa.com/>) and glucose (Sinocare Glucometer, https://en.sinocare.com) meters respectively.

Approximately 24 hours following the final dosage, animals were subjected to right and left heart catheterization and tissue collection under terminal anesthesia. The right ventricle was dissected and weighed, as was the remaining left ventricle and septum. The right lung was snap-frozen in liquid nitrogen and the left lung was isolated and perfused with 10% neutral buffered formalin (10% NBF) for 5 minutes, after which tissue was stored in 10% NBF before embedding into paraffin blocks.

**Supplemental Results**

**Supplemental Figure 1**: Blood indices in rats exposed to vehicle-normoxia (vehicle), Sugen-hypoxia (SugenH) or Sugen-hypoxia and TEPP-46 (SugenH+TEPP). Panel A shows blood lactate and panel B shows blood glucose measured at 5-6 weeks as a % change from baseline.
